# Frontline Perspectives: WIC Clinic Emergency Preparedness Following Hurricane Helene

**DOI:** 10.1007/s10995-026-04325-5

**Published:** 2026-09-24

**Authors:** Amber Riley, Ella Zhao, Sarah E. Scales, Jennifer A. Horney

**Affiliations:** 1 Department of Epidemiology, University of Delaware, Newark, USA; 2 Newark Charter High School, Newark, USA; 3 Department of Epidemiology, University of Nebraska Medical Center, Omaha, USA

**Keywords:** Disaster, Hurricane, Emergency preparedness, Social services, Women

## Abstract

**Introduction:**

The Special Supplemental Nutrition Program for Women, Infants, and Children (WIC) targets nutrition and health behaviors of high-risk populations. WIC is administered by the U.S. Department of Agriculture and provides services in 4 areas: nutrition education, breastfeeding promotion and support, healthy food access, and healthcare and social services referrals.

**Methods:**

To better understand services provided and populations served during Hurricane Helene and document personal and organizational preparedness, data were collected via an online survey. Descriptive statistics were used to characterize respondents. Odds ratios (OR) and 95% confidence intervals (CI) were calculated for associations between self-assessed preparedness and need for additional training.

**Results:**

Of 95 WIC contacts, 29 responded (30.5%). Of four key WIC services, breastfeeding support was most frequently disrupted (43.5%; n = 10), followed by health care and social service referrals (39.1%; N = 9). Respondents characterized rural residents (47.8%; n = 11) and new clients seeking services (39.1%; n = 9) as most impacted. Respondents with confidence in their organization’s ability to respond to a disaster or in their personal knowledge of disaster preparedness were less likely to want additional training. Qualitative responses described challenges providing services to evacuated clients, food security, and internet outages.

**Discussion:**

Disaster research has identified “gendered disaster vulnerability” as a challenge in emergency preparedness and response. Women and children often face intersectional risks from poverty, social marginalization, and disability in disasters. As such, the WIC workforce should be considered first responders and have access to emergency preparedness planning and training resources at the organizational and individual level.

**Supplementary Information:**

The online version contains supplementary material available at https://doi.org/10.1007/s10995-026-04325-5.

## Significance

Decades of disaster research has identified “gendered disaster vulnerability" as a challenge in emergency preparedness planning and response. As natural disasters become more frequent and severe, it is essential to identify the social services and populations most negatively affected. To inform outreach and disaster recovery programs of the needs of those most vulnerable to a disaster’s impacts, this study assessed the impact of Hurricane Helene on the emergency preparedness efforts of WIC staff and distribution of services.

**Supplementary Information:**

The online version contains supplementary material available at https://doi.org/10.1007/s10995-026-04325-5.

## Introduction

Hurricane Helene made landfall along the Florida Gulf Coast as a Category 4 storm in September, 2024. The storm moved across the Southeastern U.S., causing historic flooding, landslides, and hurricane-force winds in Georgia, South Carolina, and North Carolina (Hagen et al., 2024). Heavy rains associated with the storm led to more than 30 flash flood emergencies being declared in the southern Appalachian Mountains (Hagen et al., 2024). According to the National Oceanic and Atmospheric Administration, Helene was the deadliest hurricane to impact the U.S. since Hurricane Katrina in 2005. Federal disaster assistance was available to residents of Florida, Georgia, North Carolina, South Carolina, Tennessee, and Virginia.

The Special Supplemental Nutrition Program for Women, Infants, and Children, more commonly known as WIC, is an interventional program targeting the nutrition and health behaviors of high-risk populations in the United States (National WIC Association, n.d.). It is a short-term program administered by the U.S. Department of Agriculture’s (USDA) Food and Nutrition Service available in all 50 states, the District of Columbia, 32 Indian Tribal Organizations, as well as U.S. territories including Puerto Rico and the U.S. Virgin Islands (USDA, 2024).

Funding is allocated annually through the U.S. Senate and House Appropriations Committee (National WIC Association, n.d.). WIC provides services in 4 key areas: nutrition education, breastfeeding promotion and support, healthy food access, and screening and referrals for healthcare and social services (National WIC Association, n.d.). To participate in WIC, pregnant and postpartum women, infants, and children up to age 5 must be considered low-income and at nutritional risk (National WIC Association, n.d.). Positive health outcomes associated with WIC include but are not limited to, a reduction in the following: premature births, low birthweight infants, fetal and infant deaths, and low-iron anemia–as well as an increase in access to prenatal care, consumption of key nutrients, immunization rates, access to regular health care, and improved diet quality (National WIC Association, n.d.).

For decades, disaster research has highlighted gender inequality in both social and physical vulnerability to hazards and their impacts (Bateman & Edwards, 2002). This can take many forms. Female-headed households are more likely to have lower incomes and less access to financial and other resources (Sealza, 2019), reduced access to disaster assistance (Emrich et al., 2022), and reduced participation in the prioritization of recovery and resilience activities post-disaster (Dube & Mhembwe, 2019). Rates of gender-based violence increase in post-disaster settings across many types of disasters, which can disrupt access to social support networks and increase stress and isolation leading to coercive control (Van Daalen et al., 2022).

Eligibility for WIC is dependent on participants being classified as low-income and at risk nutritionally. As of 2022, 57% of the 6.8 million WIC participants reported an income below 100% of the Federal Poverty Guidelines–with 90% being below 185% of these guidelines (Food and Nutrition Service, 2022). Other risks such as anemia, being under or overweight, and having a history or pregnancy complications are commonly seen in pregnant, breastfeeding, and post-partum women enrolled in WIC (Food and Nutrition Service, 2022; Liu et al., 2016). Hurricanes disrupt many types of social services due to damaged infrastructure, evacuation of staff that prevents them from going to work, and other disaster-associated emigration post-hurricane. The purposes of this study include investigating WIC service providers perceptions of personal and organizational emergency preparedness and prioritizing aspects of preparedness response that could reduce the gap in vulnerable populations served by the WIC program.

## Methods

### Sample

WIC offices in states including Florida, Georgia, North Carolina, South Carolina, Tennessee, and Virginia that had counties with a federally declared major disaster from Hurricane Helene were included in the sampling frame (Federal Emergency Management Agency, 2024). Contact information for WIC offices in these counties was collected online or via telephone.

### Survey

A 19-question cross-sectional survey (Supplemental File 1) was developed based on prior research of social service agency preparedness perceptions (Horney et al., 2024). An initial email invitation was sent on June 24, 2025, followed by three weekly reminders via the Qualtrics platform (Provo, UT). Of 95 WIC contacts in disaster-impacted counties (N = 299) for whom email or telephone contact information could be found, 29 responded to the survey (Response Rate = 30.5%). The analytic sample for this survey includes 23 individuals (79.3% of respondents) who answered at least two-thirds of the questions. The survey and all related materials were reviewed and determined to be exempt under 45 CFR 46.104(d)(4) of the US Department of Health and Human Services regulations for the protection of human subjects in research by the Institutional Review Board. No identifying information was collected as part of the survey and there was no linkage between the email addresses in the invitation to complete the survey and the responses. This manuscript is not based upon clinical study or patient data.

### Measures

Respondents were asked to report personal demographics, including gender (woman, man, trans-woman, trans-man, non-binary, self-identify, prefer not say); race (American Indian or Alaska Native, Asian or Asian American, Black or African American, Native Hawaiian or Pacific Islander, white, prefer not to answer, other); and education level (less than high school, high school diploma or equivalent, vocational or technical school after high school, some college or associate’s degree, bachelor’s degree, master’s degree, professional or doctorate degree), all of which were self-reported. Occupational demographics included state of employment, job tenure in public health and specifically in WIC services, and job role prior to Hurricane Helene landfall in September 2024.

Respondents were asked to report on the services their organization provided in the 9 months since Hurricane Helene (e.g., nutrition services, breastfeeding support, health care and social or community service referrals, free healthy food distribution, and other), indicating the timeframe during which each service was affected (during Helene: September 2024, up to 6 months after Helene: October 2024–March 2025, and ongoing: April 2025—present). Groups for whom services were most likely to be disrupted after the hurricane (e.g., new clients seeking help for the first time, clients from culturally and linguistically diverse communities, clients from racially and ethnically diverse communities, clients with disabilities, clients in rural and remote communities, and clients in LGBTQIA+ relationships) were also recorded.

Finally, the survey collected data on personal and organizational preparedness perceptions. Six questions with a 3-category Likert scale response (agree, neither agree nor disagree, disagree) included having access to supports to assist family if called into work during an emergency or disaster, confidence in organization’s ability to respond, the need for assistance in organizational preparedness and response planning, the perceived importance of one’s role in a disaster or emergency, and knowledge of, and need for more training in, carrying out roles and responsibilities during a disaster or emergency.

### Analysis

Data were analyzed using SAS Studio (Cary, NC). Frequencies and percentages are reported for respondent personal and occupational characteristics. Bivariate logistic regression was used to determine the strength of association between statements of interest and reported need for additional assistance in planning for personal and organizational response to disasters or emergencies. Odds ratios (OR) and 95% confidence intervals (CI) are reported and were used to assess statistical significance between the association of statements of interest and planning support needs. Open-ended responses (See Supplemental File 1) were downloaded from Qualtrics (Provo, UT) and imported into Excel (Redmond, WA) to be inductively analyzed by the first author to identify themes.

## Results

### Respondent Personal and Occupational Characteristics

The personal and occupational characteristics of respondents in the analytic sample are outlined in Table 1. The majority of all respondents were classified as women (n = 22; 95.7%), white (n = 17; 73.9%), and holding at least a bachelor’s degree (n = 21; 91.3%). North Carolina was the most commonly represented state among respondents (n = 19; 82.6%) followed by Virginia, Tennessee, and South Carolina. Additionally, respondents reported extensive professional experience, with 78.3% reporting more than a decade of work in the field (n = 18). Finally, 82.6% of respondents reported working at a WIC clinic prior to experiencing Hurricane Helene (n = 19).

**Table 1:** Personal and occupational characteristics of survey respondents (n = 23)

Characteristic		Frequency	%
Gender	Woman	22	95.7
	Prefer not to say	1	4.3
Race/ethnicity	White	17	73.9
	Black or African American	3	13
	Asian or Asian American	1	4.3
	Native American or Alaska Native	1	4.3
	Prefer not to answer	1	4.3
Highest degree or level of education completed	Bachelor’s degree	12	52.2
	Master’s degree	8	34.8
	Some college or associate’s degree	2	8.7
	Professional or doctorate degree	1	4.3
State	North Carolina	19	82.6
	Virginia	2	8.7
	South Carolina	1	4.3
	Tennessee	1	4.3
Years in public health	> 10 years	18	78.3
	1–3 years	2	8.7
	4–5 years	1	4.3
	6–10 years	1	4.3
	< 1 year	1	4.3
Years at current site or clinic	> 10 years	14	60.9
	1–3 years	4	17.4
	4–5 years	2	8.7
	6–10 years	2	8.7
	< 1 year	1	4.3
Did you work in a WIC clinic before Hurricane Helene?	Yes	19	82.6
	No	4	17.4

### Disrupted Provision of Services

**Fig. 1:**
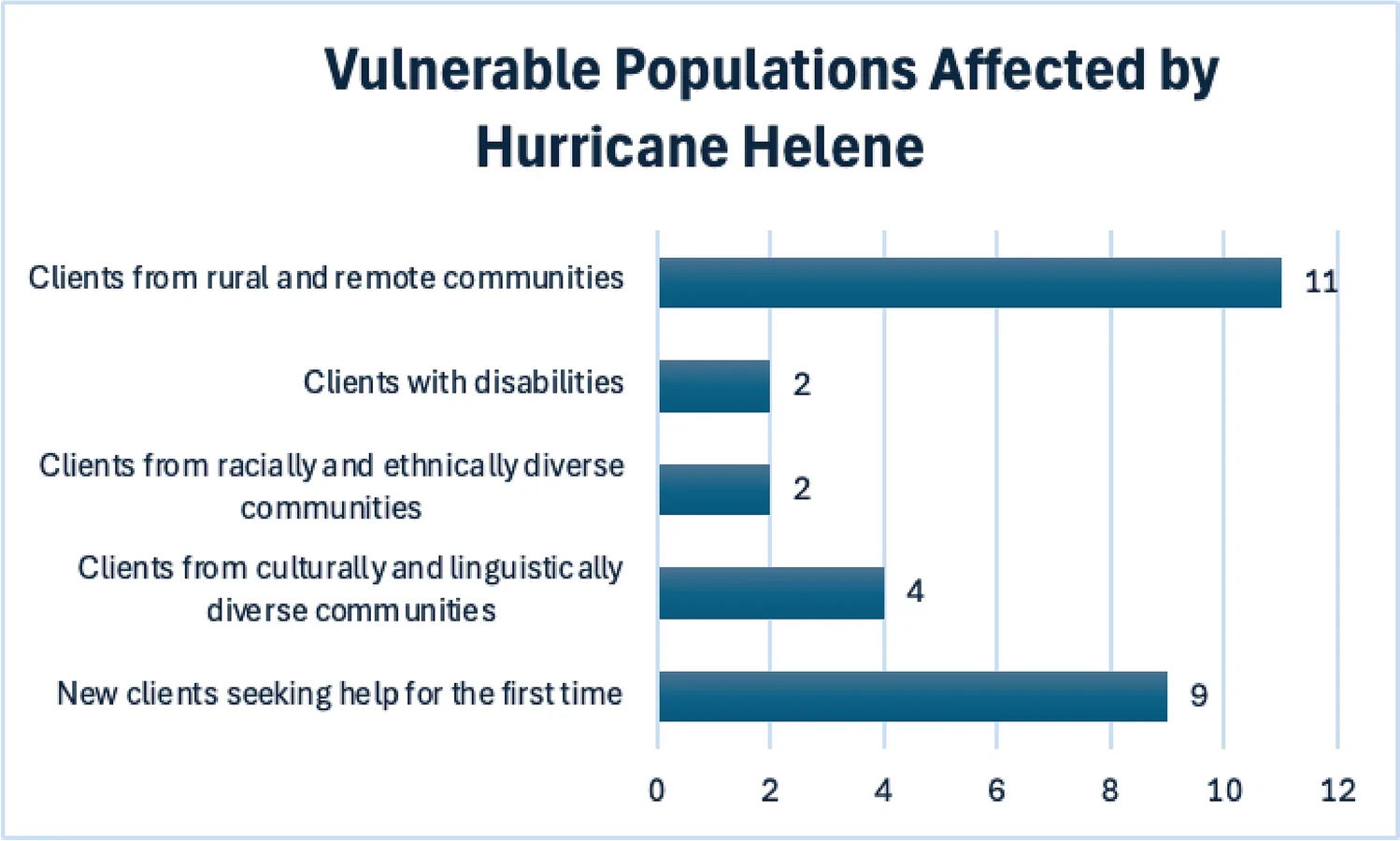
Vulnerable groups affected by Hurricane Helene (n = 23)

When asked about the disruption of the 4 key services provided by the WIC program, breastfeeding support was reported as most negatively affected during Hurricane Helene (43.5%; n = 10) (Table 2), followed by health care and social service referrals (39.1%; n = 9), which continues to experience the most impacts in the 9 months following Hurricane Helene. In terms of specific vulnerable populations, more than twice as many respondents saw greater impacts in residents of rural areas (47.8%; n = 11) (Fig. 1) and new clients seeking services in the post-hurricane time period (39.1%; n = 9). Fewer respondents identified clients from cultural and linguistically diverse communities (17.4%; n = 4), clients from racially and ethnically diverse communities (8.7%; n = 2), and clients with disabilities (8.7%; n = 2) as vulnerable populations most affected by Hurricane Helene.

**Table 2:** WIC service disruption frequencies (n=23)

Service	Disruption period		
	During Helene: September 2024	Up to 6 months after Helene: October 2024–March 2025	Ongoing: April 2025-present
Breast feeding support	43.5%	13.0%	8.7%
Health care and social/community service referrals	39.1%	21.7%	13.0%
Nutrition services	34.8%	17.4%	8.7%
Free healthy food distribution	26.1%	4.3%	8.7%
Other	8.7%	0%	0%

### Disaster and Emergency Preparedness

In bivariate analysis, both personal and occupational characteristics were not statistically significantly associated with reported personal or organizational preparedness perceptions. Individuals who responded with greater confidence in their organization’s ability to respond to a disaster or emergency (OR = 0.03; 95% CI 0.00, 0.36) (Table 3) or in their personal knowledge of disaster preparedness (OR = 0.69; 95% CI 0.13, 3.8) were less likely to want additional disaster planning or training; however, those who viewed their role in a disaster as important (OR = 1.4; 95% CI 0.20, 9.87) and those with less work experience did want additional resources (OR = 3.0; 95% CI 0.39, 23.07).

**Table 3:** Organizational confidence and personal knowledge, odds ratios and 95% confidence intervals for bivariate analysis (n = 23)

Statement	OR	95% CI
Confident in organization’s ability to respond to a disaster or emergency	0.03	0.00, 0.36*
Confident in personal knowledge of disaster preparedness	0.69	0.13, 3.8
Felt role in preparedness was important	1.4	0.20, 9.87
Wants additional resources	3.0	0.39, 23.07

^*^indicates statistically significant values

Open-ended responses characterized challenges from the effects of Hurricane Helene on professional roles and the services their agency provides, indicating that some counties saw a “small increase in (clients at) our clinic because of families moving out of affected areas.” Similarly, neighboring counties with minimal effects saw an increase in individuals being displaced and arriving for resources, in turn depleting grocery stores, with one participant noting that “shelves remained understocked for about a month.” Furthermore, many grocery stores were only able to accept cash for purchases, as internet outages prevented electronic payment, including WIC card benefits, from being used. One county reported losing their primary grocery store, with the only other grocery store accepting WIC benefits being out of the general area. While free food distribution sites were set up to help with the overwhelmed and understocked grocery stores, the increase of free infant formula handouts “without education on risk to breastmilk supply also likely impacted breastfeeding rate.” To accommodate for transportation difficulties due to road closures, virtual service delivery was attempted but proved to be challenging, both due to “limited ability to provide virtual/remote services” or loss of internet in rural areas, preventing contact completely. Recovery is also proving to be difficult, with families reporting “having a hard time getting FEMA assistance (red tape),” as well as the housing crisis which was described as “bad before but near impossible following the disaster… (forcing) many young families out of the area.”

## Discussion

### Increased Need, Fewer Services

During disasters, many types of social services–including WIC–must accommodate an influx of new clients, often with fewer resources available to serve this increased need. This is largely due to the formation of newly vulnerable populations, displacement of providers and recipients, proportionally greater impacts on those at highest risk, and interrupted service delivery (Committee on Post-Disaster Recovery of a Community's Public Health, Medical, and Social Services et al., 2015). For example, during Hurricane Maria, WIC offices were closed for several weeks due to power outages that resulted in an inability to safely store milk, largely impacting the 83% of children under 5 living in Puerto Rico who qualify for WIC services (Santaballa, 2018). Hurricane Michael, which made landfall along the Florida Panhandle in October 2018, created challenges for pregnant women seeking prenatal care (PNC), both by delaying access to care and by increasing the likelihood of inadequate PNC. In turn, this potentially increased the risk of low birthweight infants (Pan et al., 2021). In the region where this study was conducted, more than 5.9 million households lost power, with many electric providers reporting 90 to 100 percent of customers without power, some for weeks (Johnson, 2024). Although this study was cross-sectional and cannot assess temporality of births and use of WIC benefits, respondents reported that new clients seeking benefits for the first time were among the second largest group of vulnerable populations following Hurricane Helene.

Although there is relatively widespread evidence of the expanded need for social services (e.g., public health, behavioral health, public housing) post-disaster, there is less evidence focusing on how much demand for WIC services expands after disasters (Committee on Post-Disaster…, 2015). In this study, more than half of respondents observed that newer clients faced greater consequences during post-hurricane efforts aligning with published research related to help-seeking post-disaster among those with fewer resources to cope with the impacts of a disaster and limited options other than government programs like WIC (Stimpson et al., 2008). This is also consistent with research following other disasters, like Superstorm Sandy, where WIC staff reported they struggled to accept new client transfers and also dealt with challenges when transferring clients to other sites (Wemette et al., 2018). This may lead to an underestimation of need for social services post-disaster. For example, after Hurricane Michael impacted Florida in 2018, the number of pregnant women enrolled in WIC decreased, potentially due to the stress and mental toll of rebuilding, which limited the time and energy available to sign up (Pan et al., 2021). Women were also separated from their normal source of care, but left without transportation or communication resources (Harville et al., 2023).

In addition to complications regarding new client management, WIC and other services face difficulties when distributing the limited resources that are available during hurricanes and other disasters to a growing number of clients who need services or may be seeking help for the first time due to disaster impacts (Stimpson et al., 2008). Frequently, clients do not receive enough aid due to factors such as limited transportation, difficulties for WIC staff (lack of computers/electricity, locations to work, etc.), and communication issues (Wemette et al., 2018).

### Access to Nutritious Food and Nutrition Programs

In this study, more than one-third of respondents indicated that nutrition services were disrupted. Access to fresh and healthy foods was limited and disrupted by power and Internet outages, leaving some impacted counties with a single grocery store. When grocery stores become inoperable or overwhelmed, improper distribution of breast milk substitutions can occur and stores can experience a shortage of infant formula due to a larger than average demand, causing food insecurity associated with supplemental feeding (Bilgin et al., 2024; DeYoung et al., 2018 & Mudiyanslage et al., 2022). After Hurricane Maria, the closure of grocery stores and WIC offices a left many women unable to access breastfeeding supplies, visit shelter kitchens for sanitation, and refrigerate milk (due to electricity outages) (Santaballa, 2018). Deliveries were slow, food distributed by the military was nutritionally inadequate, and the water supply was unsafe (Santaballa, 2018).

Beyond needed services, post-disaster damage to transportation infrastructure and logistics systems can also limit individuals to canned and non-perishable goods available from food banks or emergency distribution sites instead of fresh food from grocery stores (Horney et al., 2026). Programs such as the Supplemental Nutrition Assistance Program (SNAP) that provide financial assistance to low-income families to supplement their food purchasing budgets, are also affected because participants are reliant on the return to pre-disaster operation of food retailers. In the aftermath of Hurricane Harvey, 92% of stores had some type of reduced access (61% closed, 35% shortened hours, and 65% had no fresh food), which hindered individuals from utilizing their benefits (Rosenheim, 2024).

### Breastfeeding Support

Breastfeeding provides essential nourishment for over 80% of U.S. born children, according to the Center for Disease Control’s National Immunization Survey-Child (CDC, 2024). During disasters, breastfeeding becomes even more important due to its nutritional, physiological, and psychological benefits (Bilgin et al., 2024). However, more than 4 in 10 respondents reported breastfeeding support was disruption by Hurricane Helene. The lack of breastfeeding support through WIC was likely compounded by limited access to nutritionists, lack of monitoring systems, and funding (Bilgin et al., 2024). Disasters impacting one’s living situation can negatively affect the ability of a woman to breastfeed due to a lack of privacy or space conducive to breastfeeding, limited fluid and calorie intake, and impaired maternal wellbeing (e.g., stress, exhaustion, trauma) (Hwang et al., 2021; Bilgin et al., 2024).

### Post-disaster Obstacles to Accessing Services and the WIC Workforce

In particular, vulnerable populations experience disproportionate obstacles when pursuing social services after a disaster. Within certain communities, preexisting conditions leave those of low socioeconomic status, individuals with prior trauma or exacerbated health issues, and other marginalized groups more vulnerable (Committee on Post-Disaster…, 2015). Female-headed households are more likely to be poor and have limited access to formal employment and credit/insurance resources and be more likely to be eligible to receive WIC benefits. Many, especially those in rural areas, struggle with poverty and substandard living conditions before disasters, making disaster recovery even more difficult (Joseph et al., 2023). Such areas have no hospitals or birth centers to offer maternity care, and many are also highly physically and socially vulnerable to the impacts of disasters (March of Dimes, 2024). Post-disaster coordination can be challenging due to the multiple systems that serve vulnerable groups. Examples of strategies that WIC providers are implementing in order to mitigate similar challenges are increased flexibility and electronic systems (eWIC). For example, some counties in Florida had no interruption to WIC operations after Hurricane Michael as benefits were uploaded to recipients’ accounts before the disaster, eliminating the need to visit offices in person (Harville et al., 2023). The flexibility of eWIC also establishes alternative benefit issuance sites and extensions for the benefits period of infants and young children (Motley, 2021). North Carolina’s public health officials specifically requested WIC flexibility (e.g., changes to vendor monitoring, waiver of certain requirements, etc.) and eWIC in Hurricane Florence as automatic issuance was shown to mitigate participation loss after Hurricane Harvey the year prior in Texas (Perdue, 2018). Such measures prove to be highly effective at times, but communication outages in disasters are fairly common, often long-lasting, and can severely disrupt the potential for success. For instance, during Hurricane Helene when power and internet outages disrupted any electronic payment methods (Marek, 2024).

Proactive preparedness is necessary to combat unforeseen challenges, so the WIC workforce must be knowledgeable and able to adapt. Public health workers generally have deep experience and extensive local knowledge about their communities. Most participants in this study have been in the field for more than 10 years, and 60.9% have worked more than 10 years at their current site or clinic. While WIC agencies are advised–and often required–to meet minimum emergency preparedness standards, it can be difficult to respond efficiently when other problems such as emergency operations coordination and confusion fueled by misinformation appear (Motley, 2021). Another concern in social services disaster response is addressing already stressful work environments that require fostering client resilience (Rahman, 2023).

### Limitations

This study has several important limitations. Although Florida was directly impacted by the landfall of Hurricane Helene, we were unable to obtain contact information for Florida WIC clinics and thus no respondents from Florida participated in the survey. Additionally, there were no responses from WIC clinics in the state of Georgia. Overall, the small analytic sample size (n=23) was a limitation that reduced statistical power. Another potential limitation is the potential for self-selection bias, which has been noted in prior disaster research and may have led those whose communities were more impacted by the disaster to be more likely to respond (Stratton, 2019). Only 5 of 23 responses (22%) used in the analysis were from non-White respondents, however, according to the Public Health Workforce Interests and Needs Survey, the public health workforce in the U.S. is predominantly (61.6%) non-Hispanic White (de Beaumont, 2024) Lastly, as a cross-sectional study, no information about the temporality of the relationship between Hurricane Helene and reported disruptions to WIC services are possible. However, since Hurricane Helene was a major disaster event, it is unlikely that respondents would not remember this exposure.

## Conclusion

Hurricane Helene affected both the services provided and the populations served by WIC across the Southeastern U.S. With breastfeeding support being most negatively impacted, there could be lasting effects on growth, development, and wellbeing of infants and children. Reported limitations of health care and social/community referrals both during and after Hurricane Helene could have limited the care people received for both chronic and new conditions, potentially worsening quality of life among the affected communities. Nutritional intake proved to be limited due to grocery store power outages and decreased food availability, also potentially impacted families receiving WIC benefits, and food insecure individuals and families more generally, disproportionately. Although clients from rural and remote communities and new clients seeking services for the first time experienced the highest reported barriers to receiving benefits, respondents working in WIC programs in the impacted areas had extensive experience and generally did not report needing additional disaster training or resources. To meet identified needs and workforce expectations, future plans may focus on the inclusion of newer and less experienced staff members in disaster planning, drills, and exercises. As natural disasters become more frequent and severe, it will be even more essential to identify the social services and populations most impacted, tailoring outreach and disaster recovery programs to meet the needs of those most vulnerable to a disaster’s impacts.

## Supplementary Information

Below is the link to the electronic supplementary material.

Supplementary file1 (DOCX 19 KB)

## Data Availability

The datasets generated during the current study are available from the Corresponding Author on reasonable request.

## Code Availability

Code is available from the Corresponding Author on reasonable request.
