# Supplementary material for: Frontline Perspectives: WIC Clinic Emergency Preparedness Following Hurricane Helene
Source: Matern Child Health J. 2026 Sep 24;30(10):1219–26. doi: 10.1007/s10995-026-04325-5 (PMC13672622; doi:10.1007/s10995-026-04325-5)
Supplement: Supplementary file 1 [file 10995_2026_4325_MOESM1_ESM.docx]

**Appendix**

*Supplemental file 1*

**WIC Preparedness & Hurricane Helene**

**Text Seen at Start of Survey:**

Thank you for your interest in completing this survey of all WIC services and clinics affected by Hurricane Helene. Your responses to this survey are anonymous and your identity will not be known to the researchers or anyone else. General findings, including tools to help your clinic or program prepare for future emergencies and disasters, may be shared with all participants who provide an email address.

**First, we would like to know a little bit about where you work and the services your agency/organization provides.**

1. In which state do you work in?
   a. North Carolina
   b. South Carolina
   c. Florida
   d. Georgia
   e. Tennessee
   f. Virginia

2. How long have you been working in the field of public health, in any capacity?

- <1 year
- 1-3 years
- 4-5 years
- 6-10 years
- >10 years

3. How long have you been in your current job site / clinic?
   - <1 year
   - 1-3 years
   - 4-5 years
   - 6-10 years
   - >10 years

4. Did you work in a WIC clinic before Hurricane Helene (September of 2024)? a. Yes

b. No

**Now we would like to learn more about the services provided during and after Hurricane Helene.**

5. During the past 9 months, in your opinion, which of these services were most frequently or severely disrupted?

|  | During Helene: September 2024 (1) | Up to 6 months after  Helene: October  2024 - March 2025  (2) | Ongoing: April 2025 - Present (3) |
| --- | --- | --- | --- |
| Nutrition services (1) | ▢ | ▢ | ▢ |
| Breastfeeding support (2) | ▢ | ▢ | ▢ |
| Health care and social/community service referrals (3) | ▢ | ▢ | ▢ |
| Free healthy food distribution (4) | ▢ | ▢ | ▢ |
| Other: (5) | ▢ | ▢ | ▢ |

6. Did you or your agency/organization see greater impacts on, or challenges to, any of the following groups as a result of Hurricane Helene? (Select all that apply)
   - New clients seeking help for the first time
   - Clients from culturally and linguistically diverse communities
   - Clients from racially and ethnically diverse communities
   - Clients with disabilities
   - Clients from rural and remote communities
   - Clients in LGBTIQ+ relationships

7. Is there anything you would like to add about how Hurricane Helene impacted your work or the services that your agency/organization provides? (OPEN ENDED / PARAGRAPH)

**A disaster is any sudden event that causes serious disruptions to the functioning of a community and exceeds its capacity to cope, including physical, weather, and climate events. In this section, please tell us a little about your personal, as well as your organization’s preparedness for a disaster.**

8. I have a person (or persons) who would take care of my family obligations in case I am called into work during a disaster or emergency.
   - Agree
   - Neither agree or disagree
   - Disagree

9. I am confident in my agency or organization’s ability to respond to a disaster or emergency.
   - Agree
   - Neither agree or disagree
   - Disagree

10. My organization needs more assistance in planning its response to a disaster or emergency.
    - Agree
    - Neither agree or disagree
    - Disagree
11. What might your organization need more assistance in? (OPEN ENDED / PARAGRAPH)

12. My role in a disaster or emergency is important.
    - Agree
    - Neither agree or disagree
    - Disagree

13. My knowledge about disaster preparedness is sufficient for carrying out my responsibilities at work during a major disaster.
    - Agree
    - Neither agree or disagree
    - Disagree

14. I would like additional training or other resources to prepare for a disaster or emergency.
    - Agree
    - Neither agree or disagree
    - Disagree

15. Is there anything you would like to add about how your agency/organization could empower clients to be more prepared for a future disaster or emergency? (OPEN ENDED / PARAGRAPH)

**Finally, we would like to learn more about the individuals who have completed the survey. Please tell us a little more about yourself.**

16. Which of the following best describes you?
    - Man
    - Woman
    - Trans-man
    - Trans-woman
    - Non-binary
    - Self-Identify: ________________________
    - Prefer not to answer

17. What would best describe your race? (Select all that apply)
    - American Indian or Alaska Native
    - Asian or Asian American
    - Black or African American
    - Native Hawaiian or Pacific Islander
    - White
    - Prefer not to answer
    - Some other race or ethnicity not listed
    - Other _______________________ *(Record other response)*

18. What is the highest degree or level of school you have completed?
    - Less than high school
    - High school diploma or equivalent (e.g., GED)
    - Vocational or technical school after high school
    - Some college of associate’s degree
    - Bachelor’s degree
    - Master’s degree
    - Professional or Doctorate degree

**Thank you for the time you spent taking this survey.** We value your feedback, so if you would like to include anything else you feel we did not ask you about that you find relevant, please include such information below. Also, **if you would like to receive a summary of the findings, please provide this email address here. Any address provided will be kept separate from your survey responses. (OPEN ENDED / PARAGRAPH)**
